# Citrate anticoagulation protocol to treat septic shock patients with liver dysfunction in CPFA extracorporeal therapy

**DOI:** 10.1186/cc11752

**Published:** 2012-11-14

**Authors:** F Ferrari, M Pozzato, T Casalicchio, S Mosca, MG Quattrone, R Cena, F Quarello, S Livigni

**Affiliations:** 1 San Giovanni Bosco Hospital Turin, Italy; 2 Bellco, CRC, Mirandola, Italy

## Background

Regional citrate anticoagulation has emerged in critically ill patients to safely treat patients with risk of bleeding. However, liver dysfunction may lead to citrate accumulation and change patients' acid-base status. In 10 years, 100 septic shock patients were treated with CPFA, an extracorporeal therapy that combines unselective plasma adsorption resin (MediaSorb) with continuous hemofiltration. Some patients suffered from liver dysfunction (total bilirubin ≥2 mg/dl) and we treated them with our citrate protocol. The aim of this study is to evaluated the safety of citrate-like anticoagulation on patients with liver dysfunction.

## Methods

Four consecutive mechanically ventilated patients (three male, one female) with septic shock and liver dysfunction were treated. Prescribed CPFA parameters were: Qb 150 ml/minute, plasma flow rate (Qp) 30 ml/minute, predilution solution (Na^+ ^136 mmol/l, citrate 10 mmol/l, citric acid 2 mmol/l) infused to keep inlet citratemia at 3 mmol/l, postdilution solution (Na^+ ^139 mmol/l, K^+ ^1.5 mmol/l, Ca^2+ ^2 mmol/l, Mg^2+ ^0.75 mmol/l, HCO_3_^- ^35 mmol/l, glucose 5.55 mmol/l) and postdilution CaCl_2 _at a rate restoring the plasma Ca^2+ ^to 1.1 mmol/l (Table 1) and adjusted according to patients' need. We evaluated five clinical parameters pre and post CPFA: pH, bicarbonate, lactate, Ca^2+^/iCa^2+^, total bilirubin.

**Table 1:** Protocol scheme for citrate-calcium chloride infusion

kg	50	55	60	65	70	75	80	85	90	95	100
Pre ml/hour	2,250	2,250	2,250	2,250	2,250	2,250	2,250	2,250	2,250	2,250	2,250
Post ml/hour	100	150	200	250	300	350	400	450	500	550	600
CaCl 10% ml/hour	4	4	4	3	3	3	3	3	2	2	2

## Results

**Figure 1:**
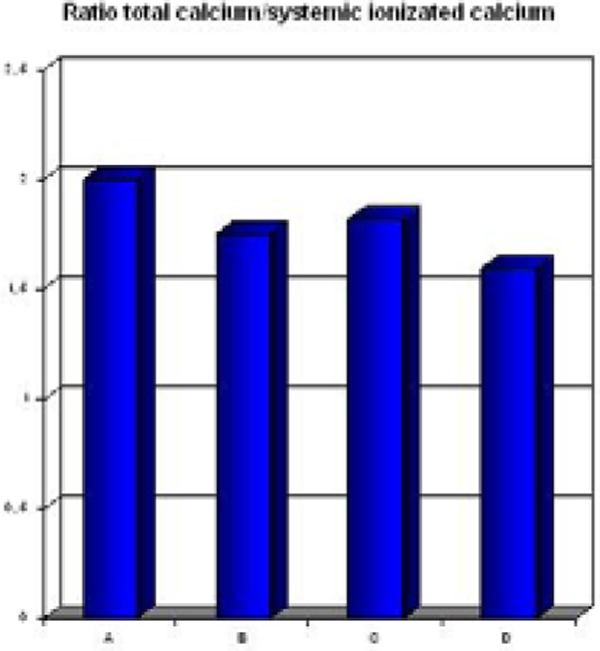
**Ca^2+^/iCa^2+ ^ratio for each patient to verify the nontoxicity of the citrate and the end of CPFA**.

**Figure 2:**
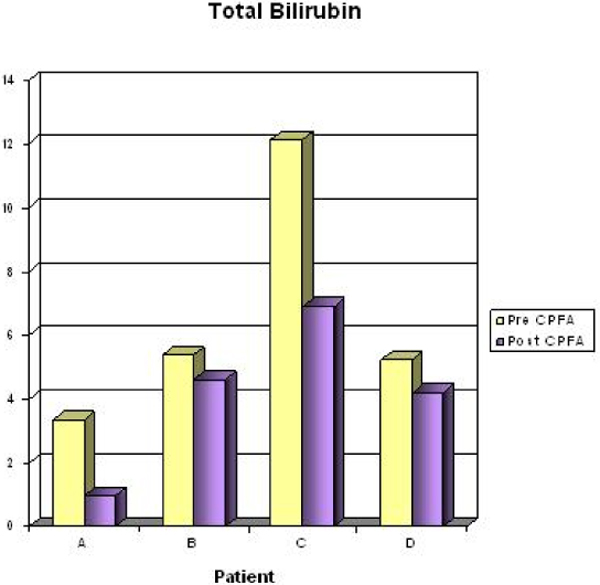
**Comparison of total bilirubin pre and post CPFA**.

Twenty-two treatments were performed accounting for 163 hours, mean duration 10.2 ± 2.1 hours, mean plasma volume of 16.4 ± 5.4 l, Qb 150 ml/minute, Qp 28.4 ± 1.8 ml/minute, and a treated plasma dose/kg body weight of 0.8 ± 0.4 l/kg. Mean CaCl_2 _10% infusion of 4.5 ± 1.3ml/hour, with citratemia, evaluated as total Ca^2+^/iCa^2+ ^ratio (Figure 1), was always <2.5 (mean 1.8 ± 0.2). For pH (mean pre 7.45 ± 0.06 vs. mean post 7.39 ± 0.07), bicarbonate (24.6 ± 3.5 vs. 24.8 ± 4.7), and lactate (2.9 ± 1.5 vs. 2.1 ± 1.6) there are no statistically significant differences between pre and post treatment. Instead we observed a decrease of bilirubin (Figure 2). Mean SOFA and SAPS II pre CPFA were 14 ± 3 and 54 ± 17, respectively. During treatment, the acid-base patients' status were kept under control with no significant electrolyte correction (Mg^2+^, K^+^, Thamesol 3.6%, NaHCO_3 _8.4%).

## Conclusion

In these four patients treated with CPFA and citrate in liver dysfunction, we have observed the absence of alteration: pH, bicarbonate, lactate and Ca^2+^/iCa^2+^. We also observed a decrease of bilirubin.

